# Supplementary material for: Bacteria from natural populations transfer plasmids mostly towards their kin
Source: Proc Biol Sci. 2019 Jun 26;286(1905):20191110. doi: 10.1098/rspb.2019.1110 (PMC6599995; doi:10.1098/rspb.2019.1110)
Supplement: Supplementary Information [file rspb20191110supp1.pdf]

nb	name	site	serotype	recipient (Rif^R)			
				R1, serotype	R1, site	R1, none	RP4, none
1	A1.1	A	8	5	2	9	7
2	A6.1	A	38	10	1	5	5
3	A6.2	A	5	9	4	14	14
4	A6.5	A	56	7	3	12	5
5	D2.2	D	8	1	6	2	2
6	D7.8	D	16	11	5	8	8
7	oc1.5	oc	56	4	8	10	10
8	oc5.6	oc	12	13	7	6	6
9	R1.9	R	5	2	10	1	/
10	R3.4	R	38	3	9	7	7
11	tm4.1	tm	16	6	12	13	13
12	tm8.6	tm	27	14	11	4	/
13	wc5.8	wc	12	8	14	11	11
14	wc6.1	wc	27	12	13	3	3

Table S1: Natural strains used and their metadata. Strain names, site of isolation and serotype information are from (Medaney et al, 2016). For each donor strain, the 4 last columns show the corresponding recipient strain for experiments shown in Figures 2 and 3, with plasmid and type of relation between donor and recipient indicated above.

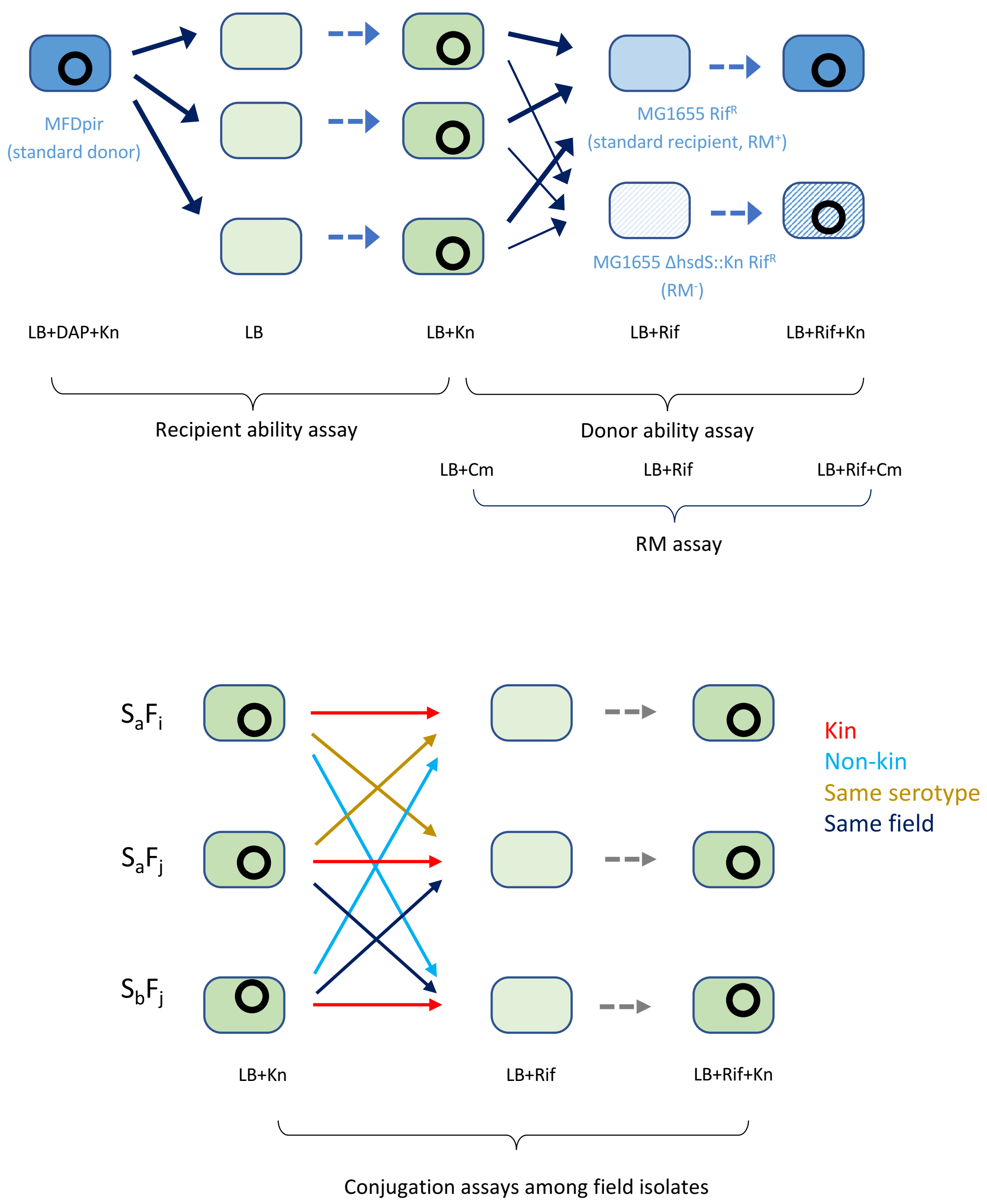

Figure S1: **Design of the conjugation assays.** K-12 laboratory strains are shown in blue, field isolates in green. Plain arrows link donor to recipient from a conjugation assay; dashed arrows point to the transconjugants obtained. Below each cell type, the selective medium on which the respective cell type density is measured is indicated. The top panel shows standard donor and recipient ability assays (Figure 1) plus RM assays (Figure 4). The bottom panel shows an example of within field collection assays (Figures 2 and 3), with strains from two serotypes (S_a and S_b) isolated from two fields (F_i and F_j), and arrows colored by type of relationship between donor and recipient.

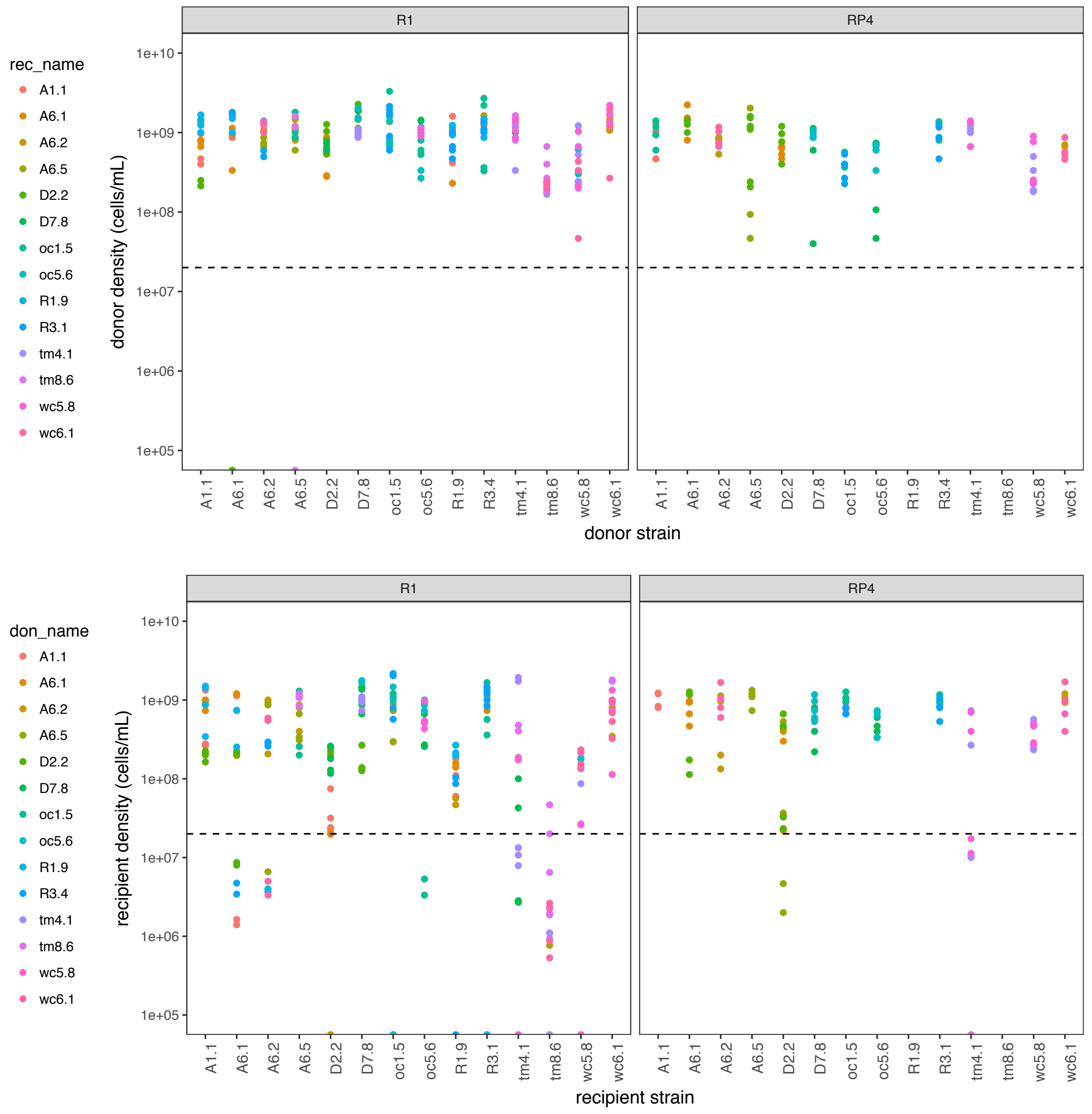

Figure S2: **Distribution of donor and recipient cell densities for assays within the field collection** (Figures 2 and 3). Each dot represents cell density (cells/mL) measured from one replicate experiment. Columns are ordered by strain measured, and color represents the identity of the interacting strain in the assay (recipient when plotting donor density and donor when plotting recipient density): variation in growth was not associated to specific competitors. The threshold density under which data were excluded (2x10^7 cells/mL) is shown as a dashed line.

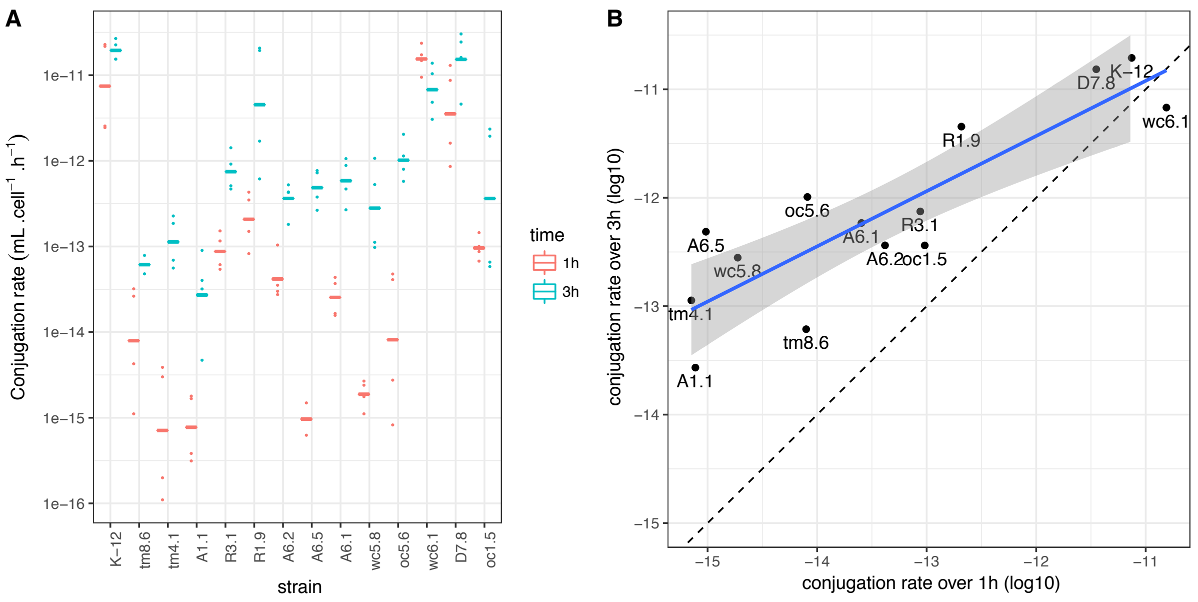

Figure S3: Effect of reduced mating times on variation in donor ability from field isolates towards K-12. In A, measured conjugation rates are shown for 1h mating (red) and 3h mating (blue), with geometric averages as lines and individual replicates as dots; in B, geometric means of conjugation rates measured for each strain after 3h are shown as a function of the respective values measured over 1h mating, showing a positive correlation but stronger increase in transfer over 3h when the initial conjugation rate is lower (the dashed line shows equal values).

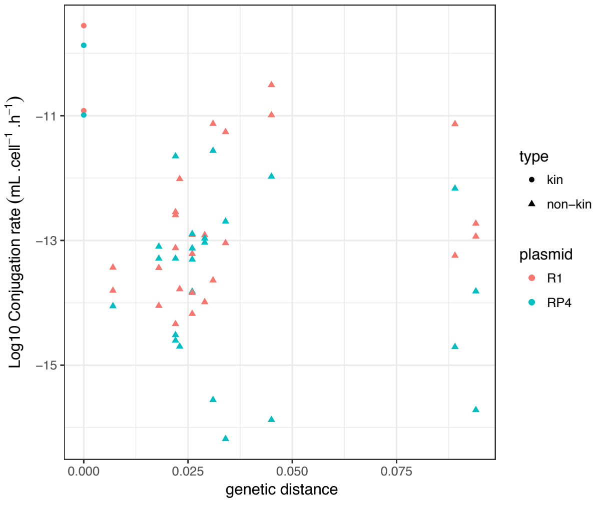

Figure S4: Conjugation rates with K-12 standard donor or recipient as a function of phylogenetic distance to K-12. Each data point represents the geometric average conjugation rate for a given donor/recipient pair, and for R1 or RP4 plasmid.

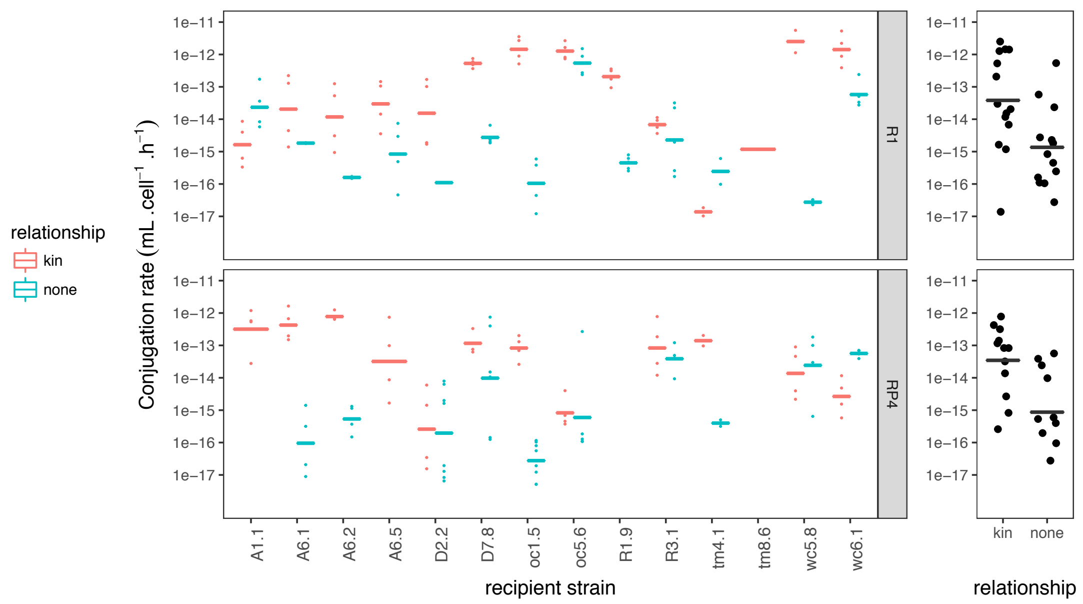

Figure S5: Transfer rates among field isolates, ordered by recipient strain. Data are the same as in Figure 2, and shown ordered by recipient isolate, with the donor isolate being the same as the recipient (kin, red) or another field isolate (non-kin, light blue, see Table S1 for donor identity). Individual replicates are shown as dots, lines are geometric means. Summary graphs at the right show average transfer per couple of strains (dots) and overall geometric means per treatment (lines).

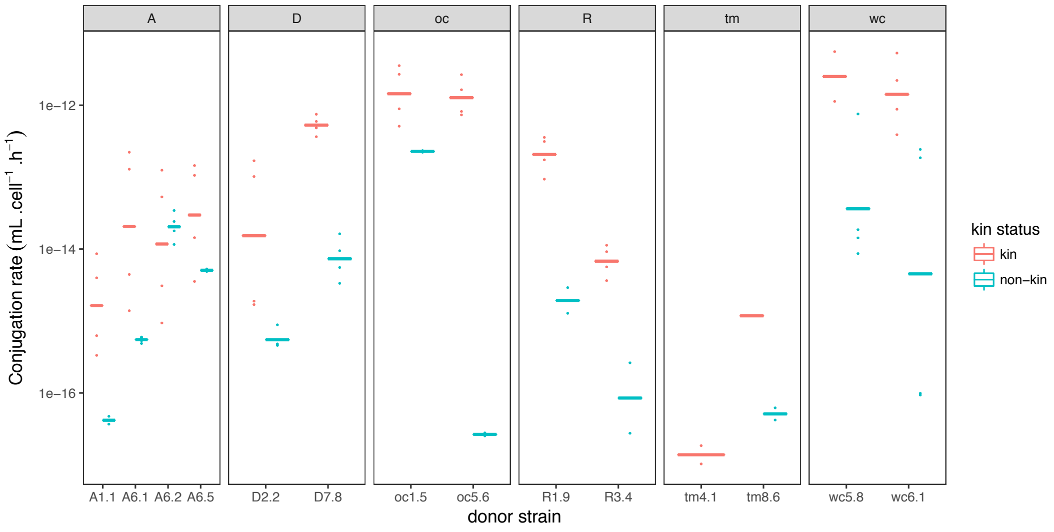

Figure S6: Conjugation rates for R1 plasmid within *E. coli* field isolates are shown here for strains isolated from the same field, subplots showing individual field sites. The donor strain is shown on the x-axis, color indicates relationship between donor and recipient (kin or non-kin) isolated from the same site. Individual replicates are shown as dots, lines are geometric means.
